# Tunable Carrier Transfer of Polymeric Carbon Nitride with Charge-Conducting CoV_2_O_6_∙2H_2_O for Photocatalytic O_2_ Evolution

**DOI:** 10.3390/nano12111931

**Published:** 2022-06-05

**Authors:** Shaohong Zang, Xiaorong Cai, Mengshan Chen, Dehong Teng, Fei Jing, Zhe Leng, Yingtang Zhou, Feng Lin

**Affiliations:** 1 Institute of Innovation & Application, National Engineering Research Center for Marine Aquaculture, Zhejiang Ocean University, Zhoushan 316022, China; shzang@zjou.edu.cn (S.Z.); caixiaorong@zjou.edu.cn (X.C.); chenmengshan@zjou.edu.cn (M.C.); z20095136223@zjou.edu.cn (D.T.); jingfei@zjou.edu.cn (F.J.); zhouyingtang@zjou.edu.cn (Y.Z.); 2 College of Chemical and Materials Engineering, Quzhou University, Quzhou 324000, China

**Keywords:** photocatalysis, water oxidation, cobalt vanadates, polymeric carbon nitride

## Abstract

Photocatalytic water splitting is one of the promising approaches to solving environmental problems and energy crises. However, the sluggish 4e^−^ transfer kinetics in water oxidation half-reaction restricts the 2e^−^ reduction efficiency in photocatalytic water splitting. Herein, cobalt vanadate-decorated polymeric carbon nitride (named CoVO/PCN) was constructed to mediate the carrier kinetic process in a photocatalytic water oxidation reaction (WOR). The photocatalysts were well-characterized by various physicochemical techniques such as XRD, FT-IR, TEM, and XPS. Under UV and visible light irradiation, the O_2_ evolution rate of optimized 3 *wt*% CoVO/PCN reached 467 and 200 μmol h^−1^ g^−1^, which were about 6.5 and 5.9 times higher than that of PCN, respectively. Electrochemical tests and PL results reveal that the recombination of photogenerated carriers on PCN is effectively suppressed and the kinetics of WOR is significantly enhanced after CoVO introduction. This work highlights key features of the tuning carrier kinetics of PCN using charge-conducting materials, which should be the basis for the further development of photocatalytic O_2_ reactions.

## 1. Introduction

Water splitting by sunlight is considered a preeminent method for converting solar energy to chemical fuels that are environmentally friendly and sustainable. The challenge of solar-to-fuel conversion is the water oxidation half-reaction (WOR) with 4e^−^, a transfer that requires a large overpotential and leads to significant losses in the overall efficiency of water splitting [1,2]. The major limits of water oxidation catalysts are (1) the poor absorption of visible light, (2) the low mobility and high recombination of charge carriers, and (3) the low efficiency of the water oxidation reaction [3,4]. Although the inspiration for water splitting comes from nature through biomimetics, photocatalysts cannot be confined to biology. To bridge the gap between the economic viability and efficiency of the WOR catalysts, tremendous efforts have been made over the years [5]. For practical applications, the catalysts should be high-performance, stable, and environmentally friendly.

Polymeric carbon nitride (PCN), a popular photocatalyst consisting of the earth-abundant elements carbon and nitrogen, is consistent with a sustainable energy economy [6,7]. The extended π-conjugated systems of PCN are beneficial to mediate a charge transfer for artificial photocatalysis. The suitable bandgap (~2.7 eV) endows PCN to overcome the endothermic character of a water splitting reaction (~1.23 eV theoretically). However, as a non-metal material, the low electrical conductivity and rapid recombination of photogenerated electron-hole pairs that lead to the photoactivity of PCN are not as good as expected [8]. Several strategies have been adopted to improve the efficiency of PCN in photocatalytic water splitting [9,10,11,12,13]. For example, morphology control is used to regulate specific surface area and adjust energy band structure [14,15,16]. Cocatalyst loading is adopted to offer active catalytic sites and facilitate the kinetics of WOR [17]. Heterojunction construction is beneficial to improve charge separation and transmission efficiency [18,19,20]. 

Transition-metal vanadates have been widely used as magnetic materials and electrode materials because of environmental friendliness, the low cost of raw materials, and excellent charge transport properties [21,22,23,24]. Since the Andrukaitis group presented the lithium intercalation of five transition metal vanadates Me_x_V_2_O_6_ (Me = Co, Ni, Mn, Cu, and Zn) [25], reports about cobalt vanadates mainly concentrate on lithium batteries, sensors, and electrocatalysts [26,27,28,29]. Cobalt vanadates acting as catalysts were explored by the Xing and Zhao group, which reported Co_3_V_2_O_8_ in electrocatalytic and photocatalytic WOR for the first time [30,31]. Pavliuk et al. designed CoV_2_O_6_/V_2_O_5_ and Co_2_V_2_O_7_/V_2_O_5_ in TiO_2_ films for electrocatalytic O_2_ evolution, which showed good stability and sustained high-current densities at relatively low overpotentials [32]. Shen et al. designed a CoV_2_O_6_–V_2_O_5_/NRGO−1 composite for electrochemical WOR with high efficiency in which CoV_2_O_6_ acted as an active site [33]. Recently, Mondal et al. established the structure-property relationship of CoV_2_O_6_ and Co_3_V_2_O_8_ for electrocatalytic O_2_ evolution, and the experimental results showed that the former was more active than the latter [34]. This is due to the more rapid etching phenomenon of the ([VO_3_]_n_)^n^^−^ in CoV_2_O_6_ than that of the discrete [VO_4_]^3−^ in Co_3_V_2_O_8_. These reports reveal that cobalt vanadates have excellent charge transfer performance, which organic polymers are lacking.

Herein, we report a CoV_2_O_6_∙2H_2_O (CoVO) hybriding with PCN by a facile immersion strategy to enhance the activity of photocatalytic water splitting to O_2_. In the CoVO/PCN hybrid system, CoVO acted as a conductive material to accelerate charge transportation and separation, which is favored for a water oxidation reaction. Moreover, the polarization curves revealed that the overpotential of the WOR was obviously decreased meaning the photocatalytic activity rate was further improved. The band gap of CoVO was narrow enough (~2.03 eV) to harvest solar light, which broadened the visible light absorption of the CoVO/PCN. The highest oxygen evolution rates of the CoVO/PCN samples were 467 and 200 μmol h^−1^ g^−1^ by UV and visible light irradiation, respectively, which were about 6.5 and 5.9 times that of the pure PCN (72 and 34 μmol h^−1^ g^−1^, respectively). The synthesis, structural characterization, physico- and electrochemical properties, and WOR performance of the hybrids will be presented in detail. 

## 2. Materials and Methods

Materials: All chemicals were used without further purification.

Urea was purchased from Shanghai Aladdin Biochemical Technology Co., Ltd. (Shanghai, China). NH_4_VO_3_, TMCl_x_·nH_2_O (Fe, Co, Ni, Cu, Mn) were purchased from Sinopharm Chemical Reagent Co., Ltd. (Shanghai, China). All the reagents were of analytical grade and were used without further purification.

### 2.1. Synthesis of PCN

PCN was synthesized by a traditional thermal polymerization strategy following our previous report [35]. Urea (10 g) was put into a crucible and annealed at 550 °C for 2 h in the air. The obtained yellow powder was PCN.

### 2.2. Synthesis of Transition-Metal Vanadates (Fe, Co, Ni, Cu, Mn)

Transition-metal vanadates were synthesized by hydrothermal method [31,36]. NH_4_VO_3_ (4 mmol) was dissolved and stirred in deionized water (80 mL) at 80 °C. Then, TMCl_x_·nH_2_O (0.8 mmol) was added and the solution was stirred and heated until a solution was obtained. The solution was transferred into a Teflon-lined autoclave and reacted at 180 °C for 12 h. After the reaction was over, the sample was centrifugated and washed with deionized water and ethanol at least three times. The solid was dried at 80 °C overnight.

### 2.3. Synthesis of CoVO/PCN

The CoVO/PCN photocatalysts were obtained by an immersion method [20]. The as-prepared PCN (500 mg) and CoVO (20 mg) were immersed separately in deionized water (2 mL). The two suspensions were placed in an ultrasound machine for 20 min. Then, different amounts of CoVO suspension were added to the above PCN suspension with continuous ultrasound. After evaporating and drying in an oven at 80 °C overnight, the final obtained solid was CoVO/PCN. The as-synthesized solids were marked as x *wt*% CoVO/PCN (x = 1, 2, 3, 5, and 10).

The other TMVO/PCN (TM: Fe, Co, Ni, Cu, Mn) samples were obtained using the same method.

### 2.4. Characterization

X-ray diffraction (XRD) measurements to manifest the crystal structure of photocatalysts were carried out on a Bruker D8 Advance diffractometer with CuKa1 radiation. Transmission electron microscopy (TEM) to observe morphological characteristics of photocatalysts was performed on an FEI Tencai 20 microscope. X-ray photoelectron spectroscopy (XPS) to analyze the chemical state of photocatalysts was obtained using a Thermo ESCALAB250 instrument. Room-temperature photoluminescence spectra (PL) were obtained by an Edinburgh FI/FSTCSPC 920 spectrophotometer. UV-vis diffuse reflection spectra (UV/Vis DRS) to analyze optical properties of photocatalysts were operated by a Varian Cary 500 Scan UV/Vis system. Electrochemical tests were performed on a Shanghai Chenhua Electrochemical Workstation. The three-electrode cells contained a working electrode, a counter electrode (Pt plate), and a reference electrode (Ag/AgCl electrode).

### 2.5. Photoactivity for Water Oxidation Reaction

Photocatalytic WOR occurred in a Pyrex top-irradiation reaction vessel. The reaction temperature in the photocatalytic system was kept at room temperature by circulating condensed water. The light source for the experiment is a 300 W Xeon lamp. The working current of the lamp is 15 A (CEL-HXF300). Generated gases in the reaction were tested by gas chromatography (a thermal conductive detector and a 5Å molecular sieve column) in which the carrier gas was Argon. In this system, the catalyst (50 mg) was well-dispersed in distilled water (100 mL) and added electron scavenger AgNO_3_ (0.17g) and pH buffer agent La_2_O_3_ (0.2 g). Before being irradiated, the air in the reaction solution was removed by evacuating several times.

## 3. Results and Discussion

**Figure 1:**
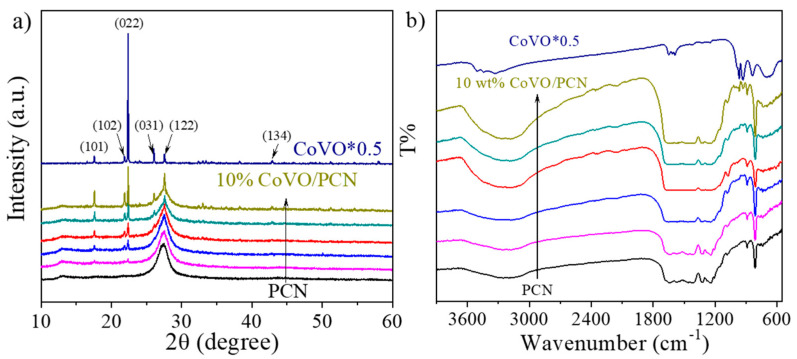
(**a**) Powdered XRD patterns; (**b**) FT-IR spectra of x *wt*% CoVO/PCN samples (black: PCN, violet: 1 *wt*%, blue: 2 *wt*%, red: 3 *wt*%, green: 5 *wt*%, beige: 10 *wt*%, navy: CoVO*0.5, Peak intensity decreased by 0.5 times).

The powered XRD patterns of the x *wt*% CoVO/PCN hybrids are shown in Figure 1a. There are six individual peaks located at 2θ = 17.6, 21.8, 22.4, 26.0, 27.5, and 42.8°, corresponding to (101), (102), (022), (031), (122), and (134) of CoV_2_O_6_∙2H_2_O [36]. No impurity peaks of the prepared materials could be observed, illustrating that CoV_2_O_6_∙2H_2_O was well-crystallized with high purity. The reflection at 2θ = 13.0° of PCN corresponds to an in-plane repeating motif along the (100) direction and the peak at 2θ = 27.4° is the stacking of the CN-conjugated layers along the (002) direction [10]. The characteristic diffraction peaks of the CoVO/PCN samples match well to both CoVO and PCN, indicating that CoVO is attached to the surface of PCN. However, the relative intensity between the (101) and (022) planes drastically changed from CoVO to 10% CoVO/PCN. The intensification of the (101) and (022) peaks changed after the immersion treatment but their positions were not shifted. In addition, the (102), (031), and (122) peaks of CoVO were also intensified [37]. The main reason is that the secondary development of the crystal surface was promoted by heat treatment during the immersion process and the conclusion can be confirmed by the XRD of CoVO before and after immersion (Figure S1a).

The FT-IR spectra of the x *wt*% CoVO/PCN samples as well as those of pure CoVO and PCN are shown in Figure 1b. The peaks at 809 cm^−1^ and 1200 to 1600 cm^−1^ are corresponding to the s-triazine ring vibration and the vibration bands of the aromatic C–N heterocycles of PCN. The strong characteristic peaks located at 930 and 970 cm^−1^ of CoVO can be obviously observed in the hybrids with the content of CoVO increasing. The FT-IR spectra again proved that the CoVO/PCN hybrids were successfully constructed. The typical characteristic peaks of PCN shown in the XRD patterns and FT-IR spectra mean that the main graphitic structure of PCN does not change and the CoVO/PCN hybrids are successfully constructed [20].

**Figure 2:**
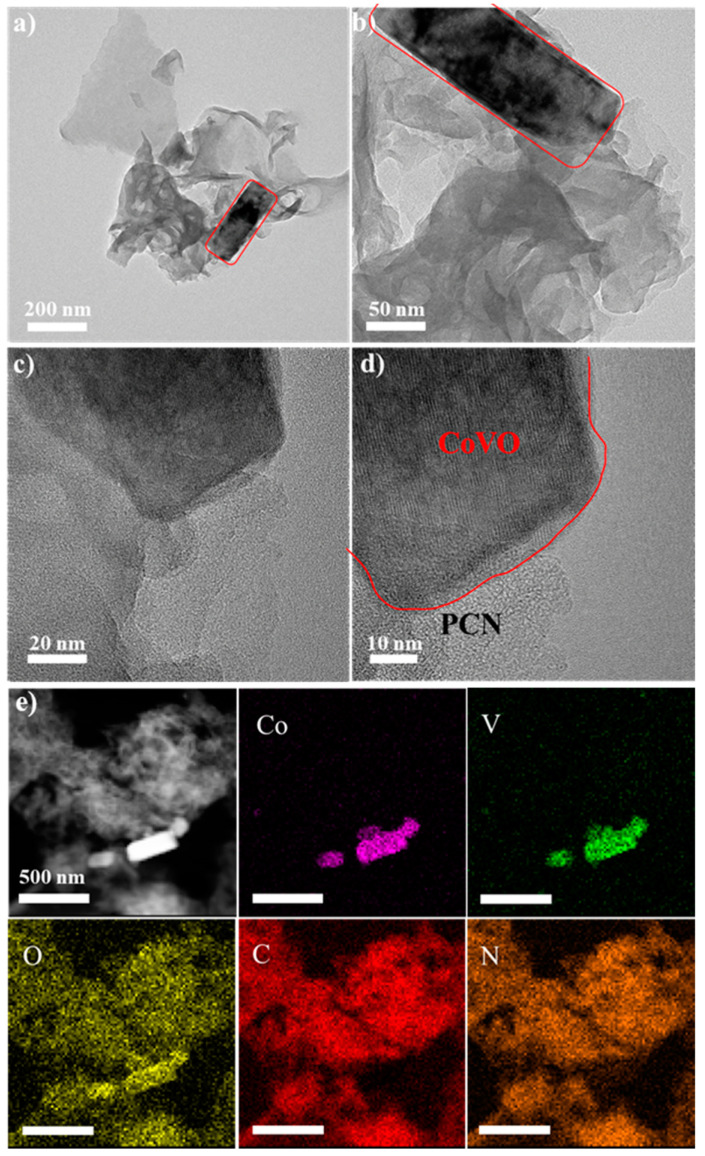
(**a**–**d**) TEM images; (**e**) HRTEM mapping of 3 *wt*% CoVO/PCN sample.

TEM images were carried out to observe the morphology characteristics of the 3 *wt*% CoVO/PCN composite. As shown in Figure 2a–d, the crystal CoVO was loaded on the surface of the typical silk nanosheets of PCN. Evident crystalline could be viewed on the surface of the two-dimensional polymer directly. The TEM images of the CoVO/PCN solid showed that these interactions led to the formation of an intact interface between CoVO and PCN. Since the surface of the graphitic carbon nitride was fully intact with cobalt vanadate, the charge transfer between the vanadate semiconductor and polymeric carbon nitride will be unobstructed. Both TEM elemental mapping (Figure 2e) and EDX analysis (Figure S2) of CoVO/PCN showed Co, V, O, and C, N elements visually belonging to CoVO and PCN. The coexistence of CoVO lattices and amorphous PCN observed in Figure 2c,d illustrate that a tight heterojunction between CoVO and PCN was formed in the hybrid [38]. Additionally, the BET-specific surface areas of PCN and 3 *wt*% CoVO/PCN were 62 and 56 m^2^ g^−1^ (Figure S1b), respectively. The slight change in the BET-specific surface area is mainly because of the weak agglomeration of PCN during the immersion process. The TEM and EDX analysis illustrate that CoVO and PCN are successfully combined and the main structure of PCN does not change during the immersion process. The result is consistent with that of the analysis of the XRD and FT-IR spectra.

**Figure 3:**
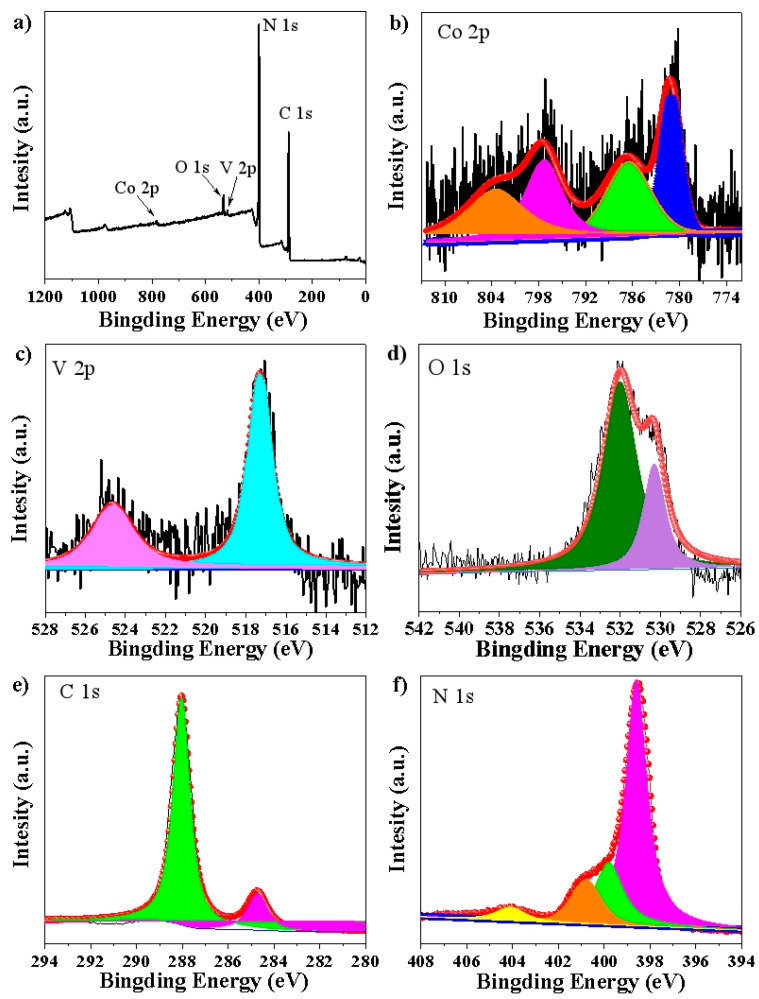
High-resolution XPS spectra of 3 *wt*% CoVO/PCN samples (**a**) survey; (**b**) Co 2p; (**c**) V 2p; (**d**) O 1s; (**e**) C 1s; (**f**) N 1s.

XPS analysis was attempted to determine the surface composition and chemical nature of the 3 *wt*% CoVO/PCN samples. Co 2p, V 2p, O 1s, C 1s, and N 1s could be observed in the complete survey spectrum (Figure 3a), which is in accordance with the EDX analysis. The Co 2p spectrum in Figure 3b is described as two peaks of Co 2P_2/1_ at 797.3 eV and Co 2P_2/3_ at 781.1 eV, with additional satellite peaks situated at 787.1 eV and 803.5 eV [36]. The V 2p spectrum in Figure 3c is located at 524.6 eV and 517.2 eV corresponding to the V 2p_1/2_ and V 2p_3/2_ [36,38]. The O 1s spectrum is shown in Figure 3d and can be split into three peaks at binding energies of 530.3 and 532.0 eV, which were attributed to the Co–O bond and the oxygen in the surface hydroxyl groups (C–OH) [28]. Furthermore, a high-resolution XPS analysis of C 1s and N 1s belonging to PCN was also carried out, as seen in Figure 3e. The peaks of C 1s can be divided into two peaks at binding energies of 288.0 and 284.7 eV, respectively. The former was assigned to the sp^2^ hybridized carbon of the triazine ring (N–C=N) of PCN and the latter was assigned to the C=C group of PCN [35,39]. In Figure 3f, four peaks of N 1s can be observed. The peak at 404.0 eV could have resulted from the charging effects or positive-charge localization in the heterocycles. The peak at 400.8 eV was due to the surface uncondensed C–N–H groups. The peak at 399.8 eV belonged to the tertiary N–(C)_3_ groups, whereas the peak at 398.5 eV corresponded to sp^2^-hybridized nitrogen (C=N–C) in aromatic triazine rings [39,40]. The last two forms of N and the sp^2^–C together constituted the heptazine heterocyclic melon ring (C_6_N_7_) units of polymeric carbon nitride [41]. Notably, decreased binding energy means higher electron cloud density. Therefore, the XPS data in Figure S3 revealed that there was a strong interaction between PCN and CoVO, and the electrons were moved from CoVO to PCN [20].

**Figure 4:**
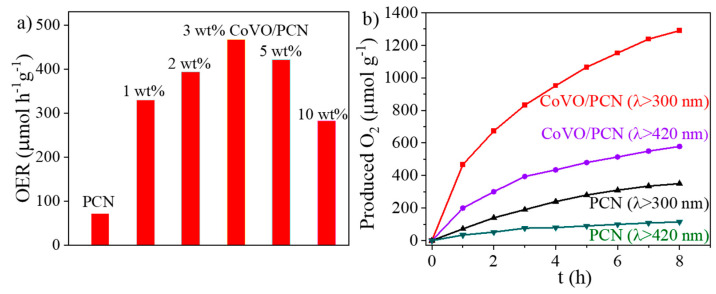
Photocatalytic O_2_ evolution curves of (**a**) x *wt*% CoVO/PCN samples under UV irradiation (λ > 300 nm); (**b**) eight-hour continuous test of 3 *wt*% CoVO/PCN sample under UV (λ > 300 nm) and visible light irradiation (λ > 420 nm).

The photocatalytic performance of the as-prepared CoVO/PCN samples was examined by a water oxidation reaction. In the system, AgNO_3_ acts as an electron scavenger to short-cut the reduction side. The oxygen evolution rate of the composites in Figure 4a was much higher than that of pure PCN. Particularly, the 3 *wt*% CoVO/PCN sample showed the best performance of O_2_ evolution rate of 467 µmol h^−1^ g^−1^, whereas PCN is 74 µmol h^−1^ g^−1^ irradiated by UV light(λ > 300 nm). A series of reference experiments were operated under the same conditions. When the CoVO/PCN was not added to the system, no O_2_ gas was detected. These results illustrate that the WOR is driven by the CoVO/PCN photocatalyst. When the amount of CoVO in the hybrids increased to 10 wt%, the activity of the WOR decreased to 282 µmol h^−1^ g^−1^. Although it is still much higher than that of PCN, modification with excessive CoVO is unfavorable. This could be attributed to the light-blocking effect [42].

Furthermore, a long time test of 3 *wt*% CoVO/PCN sample was carried out with UV (λ > 300 nm) and visible light (λ > 420 nm) irradiation. The amounts of evolved O_2_ reached 1290 and 578 µmol g^−1^ of 3 *wt*% CoVO/PCN and 350 and 115 µmol g^−1^ of pristine PCN after 8 h persistent irradiation, as seen in Figure 4b. O_2_ can be continuously produced over the CoVO/PCN photocatalyst during light irradiation but the O_2_ amount per hour is obviously reduced. The O_2_ amount of the recycled sample shows a slightly reduced rate (Figure S4) because the amount of the electron scavenger AgNO_3_ was reduced. Moreover, Ag^+^ was reduced to Ag and loaded on the surface of PCN in the reaction, which hinders light absorption. The conclusion could also be confirmed by the XRD and DRS spectra of the recycled catalyst (Figure S5). The major structures of the CoVO/PCN samples showed no obvious changes except for the appearance of Ag peaks, which illustrated that the hybrids were stable during the photocatalytic process [43]. Other common transition metal vanadates (TMVO, TM: Fe^3+^, Ni^2+^, Cu^2+^ and Mn^2+^) obtained by a similar hydrothermal method were tested to drive the WOR under the same experimental conditions. The same amount of TMVO was adopted to combine with PCN (named TMVO/PCN). Although all the metal vanadates can enhance the activity of water oxidation to O_2_, the 3 *wt*% CoVO/PCN sample is still the best one (Figure S6). This may be due to the different chemical states of cobalt (Co^2+^, Co^3+,^ and Co^4+^), which facilitate the redox transformation, thus leading to the optimal performance in photocatalytic WOR [44].

**Figure 5:**
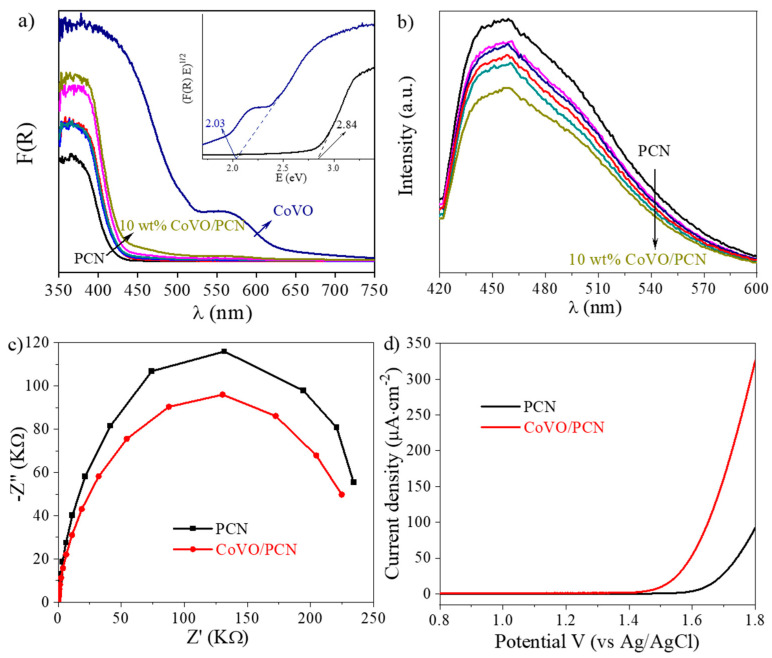
(**a**) UV-vis DRS spectra (inset: Taus plots); (**b**) PL spectra of x *wt*% CoVO/PCN samples; (**c**) EIS Nyquist curves; (**d**) Polarization curves of 3 *wt*% CoVO/PCN and PCN samples.

The photoactivity of WOR depends on visible light absorption, the photogenerated charge transfer rate, and recombination. So, UV-vis DRS, room temperature photoluminescence (PL), and photoelectrochemical analysis were adopted to further investigate the reasons for the enhanced photoactivity in WOR. In Figure 5a, the light absorption edge of CoVO/PCN samples showed a red-shift with an increasing amount of CoVO [12]. The optical absorption band edge was extended from 430 nm to 650 nm. The construction of the CoVO/PCN heterojunction is not only good for realizing a wide spectrum of visible-light absorption but also potentially promotes the production of photogenerated electron-hole pairs. In Figure 5b, the PL spectra of the CoVO/PCN samples were remarkably weaker than those of PCN, indicating that the recombination rate of the charge carriers in CoVO/PCN has been effectively restrained [39]. It is clearly demonstrated that charge separation and transfer in the CoVO/PCN samples are promoted, which is one of the reasons for the improved photoactivity.

The electrochemical impedance spectroscopy (EIS) was implemented to explore the interfacial resistance between the photocatalyst electrode and the electrolyte solution. In Figure 5c, an obviously smaller interfacial resistance is received than that of pure PCN, which means the resistivity of 3 *wt*% CoVO/PCN is much smaller. The decreased impedance of the semiconductor is beneficial to the charge transfer and promotes the oxygen evolution kinetics [8,45]. The positive current in the 1.4–1.8 V versus Ag/AgCl (V vs. RHE) in the polarization curves in Figure 5d can be attributed to the oxygen evolution. The much-reduced overpotential of CoVO/PCN means that the excessive droving potentials of the photocatalytic water oxidation kinetics can be avoided [44,46]. The experimental results reveal that CoVO in the role of conductive material in the hybrids performs very well in photocatalytic WOR. To conclude, the excellent performance of the CoVO/PCN samples in the photocatalytic WOR is a result of the superior visible light absorption, faster charge transfer, fewer charge recombinations, and the smaller overpotential of WOR than that of undecorated PCN.

**Scheme 1:**
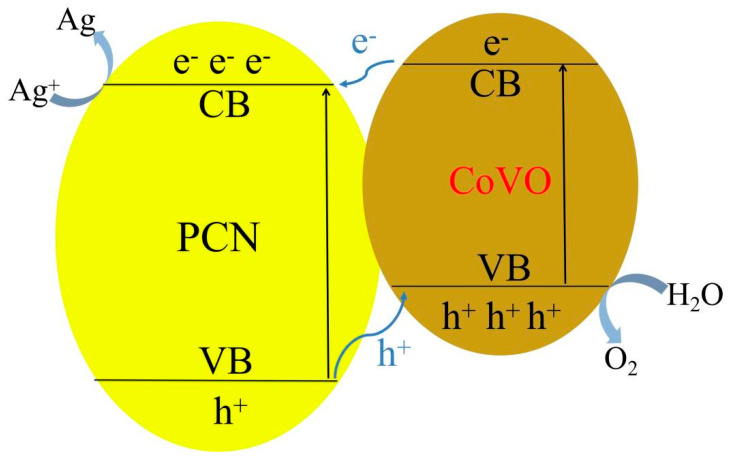
Possible charge carrier transfer route between PCN and CoVO.

The band structure of CoVO was determined by Mott-Schottky plots and DRS spectra (Figure S7). Based on the experimental analysis, a possible photocatalytic mechanism of CoVO/PCN samples in WOR was proposed in Scheme 1. Irradiated by solar light, the photo-excited electrons generated from the conduction band of CoVO transfer to the PCN side and are obtained by Ag^+^. Meanwhile, the holes generated at the valence band of PCN moved to the CoVO side and reacted to O_2_. The conclusion is in accordance with the results of the XPS. Therefore, the construction of the CoVO/PCN heterojunction cannot only be conducive to the charge transfer but also restrains the charge recombinations and finally improves the photoactivity in WOR [47].

## 4. Conclusions

In summary, CoVO/PCN was constructed by a simple immersion method and performed significantly improved photoactivity for the water oxidation reaction. With the introduction of CoVO, the visible light absorption of PCN is broadened. Furthermore, the good charge-conducting property of CoVO not only accelerates the transmission and separation of photogenerated electron-hole pairs but also restrains the recombination of electron-hole pairs in the hybrids. Moreover, it reduces the overpotential of photocatalytic water oxidation to O_2_, which is conducive to facilitating the reaction rate. The optimal photocatalytic O_2_ evolutions reach 467 and 200 μmol h^−1^ g^−1^ under UV and visible light irradiation, which are, respectively, a 6.5- and 5.9-fold enhancement of that of the undecorated PCN. This work explores the role of cobalt vanadates in photocatalytic water splitting and provides new ideas for the design of efficient photocatalysts.

## Data Availability

Not applicable.

## Supplementary Materials

The following supporting information can be downloaded at: https://www.mdpi.com/article/10.3390/nano12111931/s1, Figure S1: (a) XRD pattern of CoVO before and after immersion, (b) BET specific surface areas of PCN and 3 *wt*% CoVO/PCN samples; Figure S2: EDX mapping of the 3 *wt*% CoVO/PCN sample; Figure S3: High-resolution XPS spectra of pure CoVO, PCN and the 3 *wt%* CoVO/PCN samples; Figure S4: Recycling OER ability of 3 *wt*% CoVO/PCN; Figure S5: (a) XRD spectra and (b) DRS spectra of the recycled CoVO/PCN sample; Figure S6: Photocatalytic O_2_ evolution curves of 3 *wt*% TMVO/PCN samples (TM: Fe, Co, Ni, Cu, Mn); Figure S7: (a) Mott-Schottky plots of CoVO; (b) electronic band structure of the PCN and CoVO.
