# Supplementary material for: Tunable Carrier Transfer of Polymeric Carbon Nitride with Charge-Conducting CoV2O6∙2H2O for Photocatalytic O2 Evolution
Source: Nanomaterials (Basel). 2022 Jun 5;12(11):1931. doi: 10.3390/nano12111931 (PMC9182530; doi:10.3390/nano12111931)
Supplement: Supplementary file 1 [file nanomaterials-12-01931-s001.zip › nanomaterials-1730411-supplementary.pdf]

Supplementary Materials

Tunable Carrier Transfer of Polymeric Carbon Nitride with Charge−Conducting $CoV_2O_6{\cdot}2H_2O$ for Photocatalytic O_2 Evolution

Shaohong Zang [1], Xiaorong Cai [1], Mengshan Chen [1], Dehong Teng [1], Fei Jing [1], Zhe Leng [1,*], Yingtang Zhou [1] and Feng Lin [2,*]

[1] Institute of Innovation & Application, National Engineering Research Center For Marine Aquaculture, Zhejiang Ocean University, Zhoushan 316022, China; shzang@zjou.edu.cn (S.Z.); caixiaorong@zjou.edu.cn (X.C.); chenmengshan@zjou.edu.cn (M.C.); z20095136223@zjou.edu.cn (D.T.); jingfei@zjou.edu.cn (F.J.); zhouyingtang@zjou.edu.cn (Y.Z.)

[2] College of Chemical and Materials Engineering, Quzhou University, Quzhou 324000, China

* Correspondence: lengzhe@zjou.edu.cn (Z.L.); 39083@qzc.edu.cn (F.L.); Tel.: +86-0580-2262589 (Z.L.)

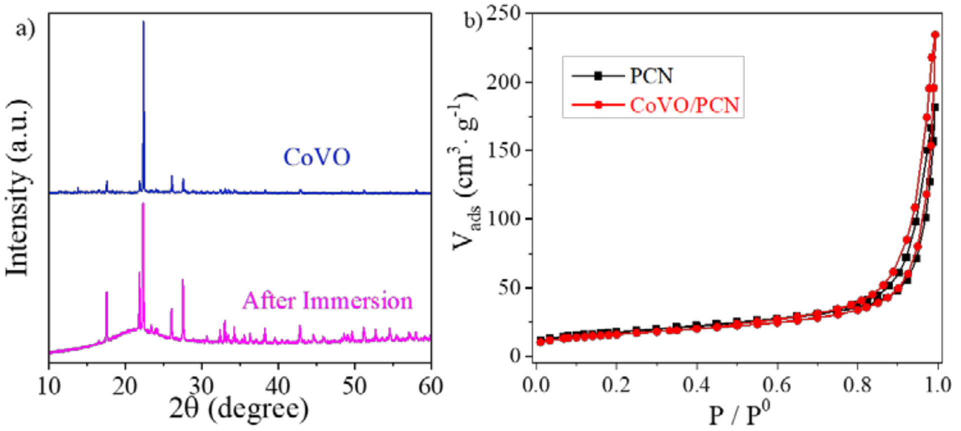

Figure S1. (a) XRD pattern of CoVO before and after immersion, (b) BET specific surface areas of PCN and 3 *wt*% CoVO/PCN samples.

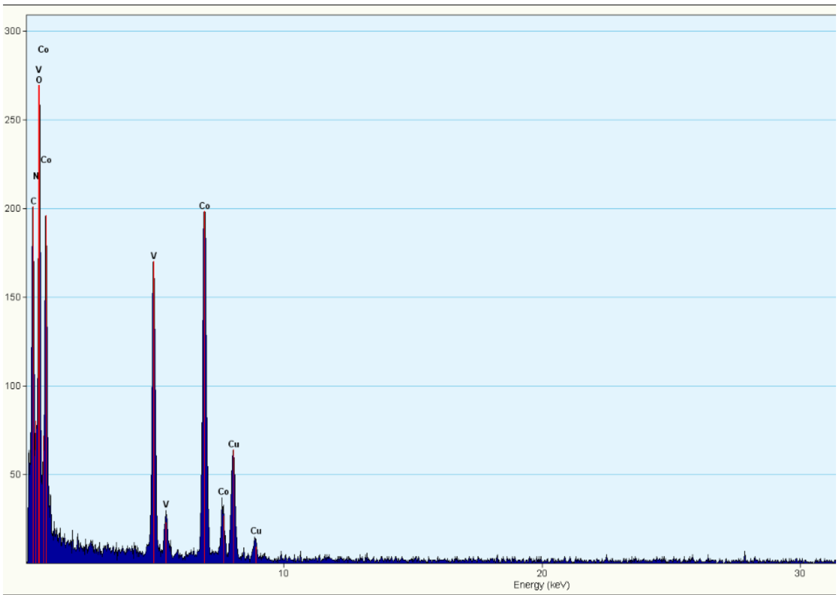

Figure S2. EDX analysis of 3 *wt*% CoVO/PCN sample.

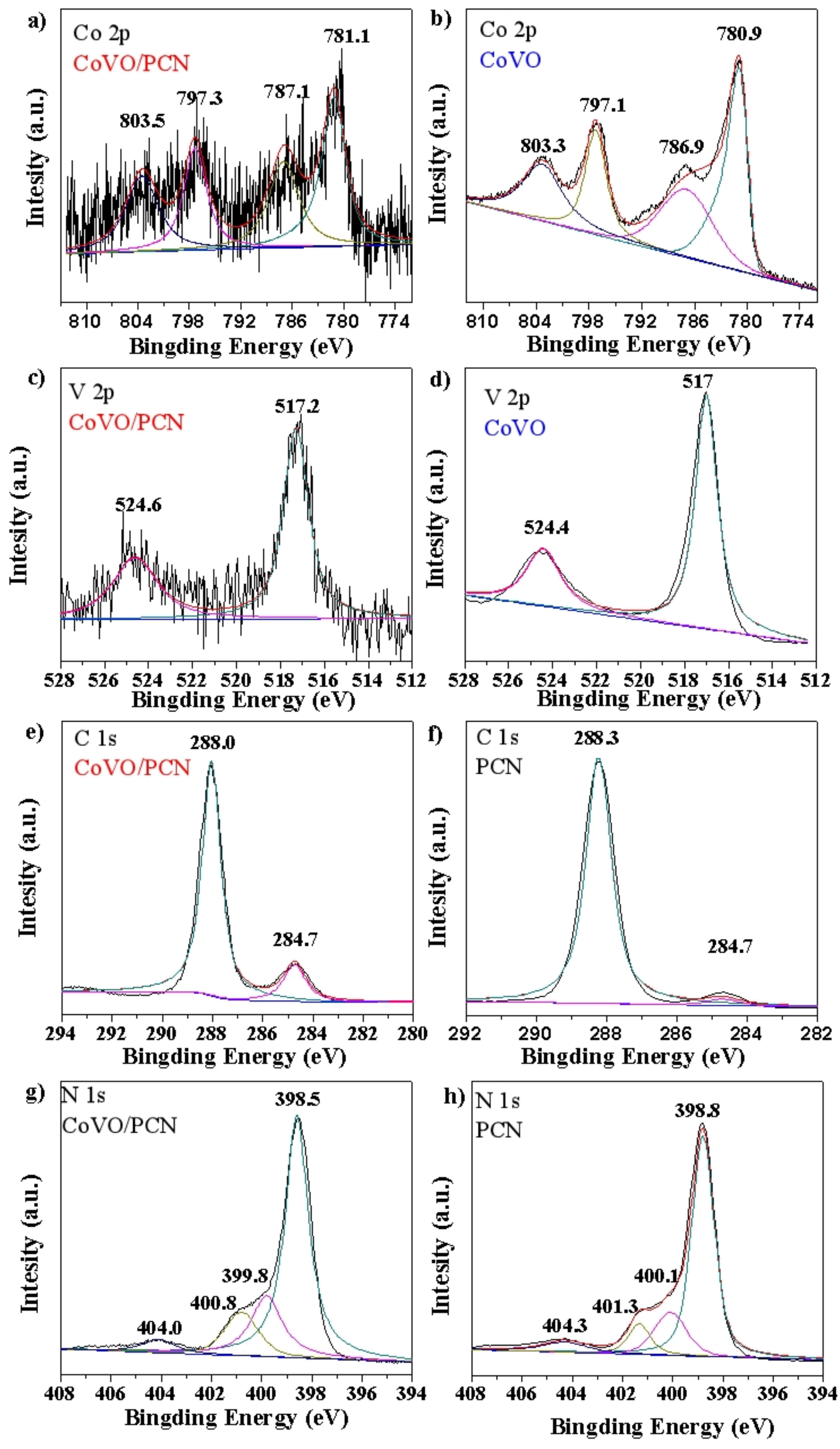

Figure S3. High-resolution XPS spectra of pure CoVO, PCN and the 3 *wt*% CoVO/PCN samples.

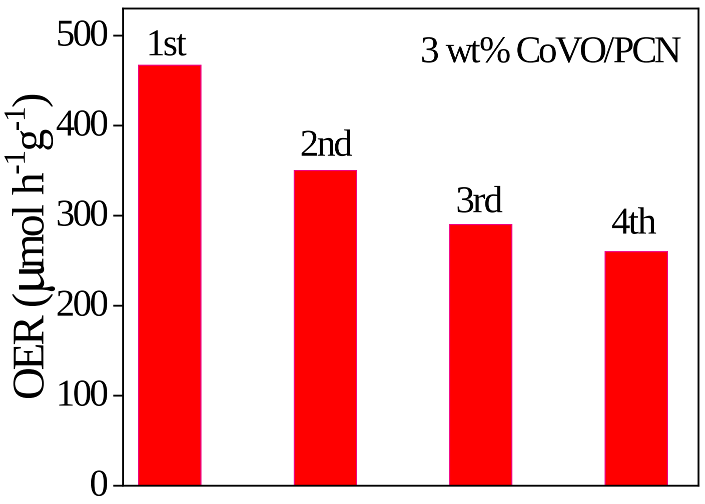

Figure S4. Recycling OER ability of 3 *wt*% CoVO/PCN.

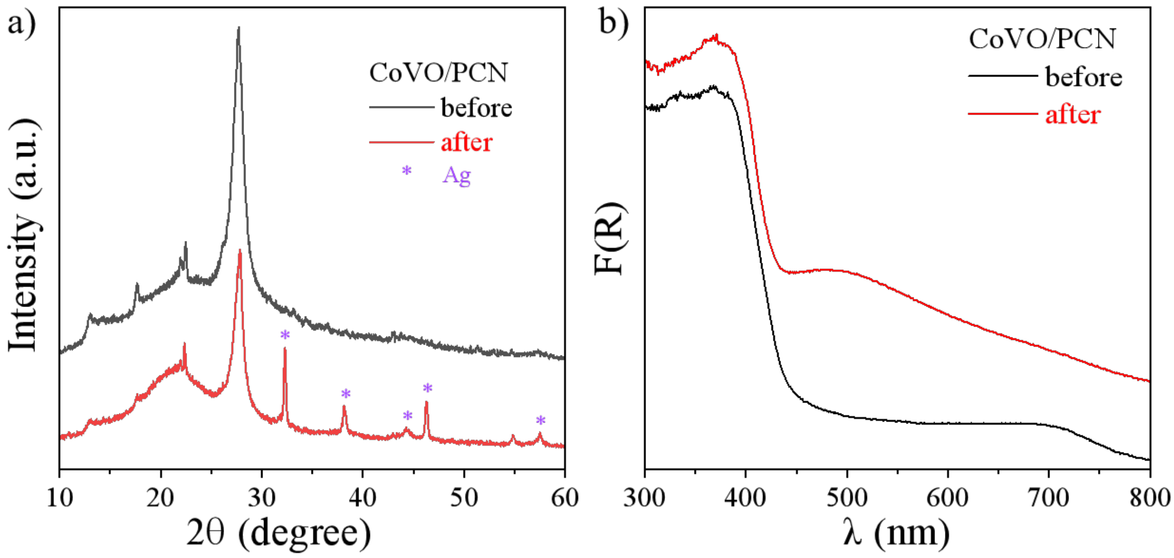

Figure S5. (a) XRD spectra and (b) DRS spectra of the recycled CoVO/PCN sample.

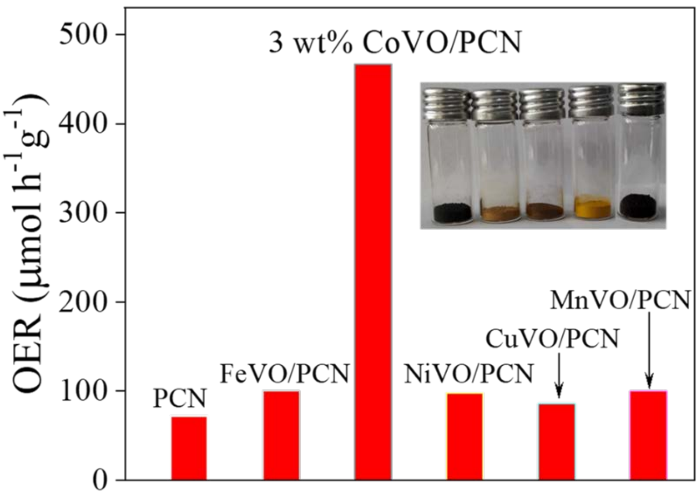

Figure S6. Photocatalytic O_2 evolution curves of 3 *wt*% TMVO/PCN samples (TM: Fe, Co, Ni, Cu, Mn).

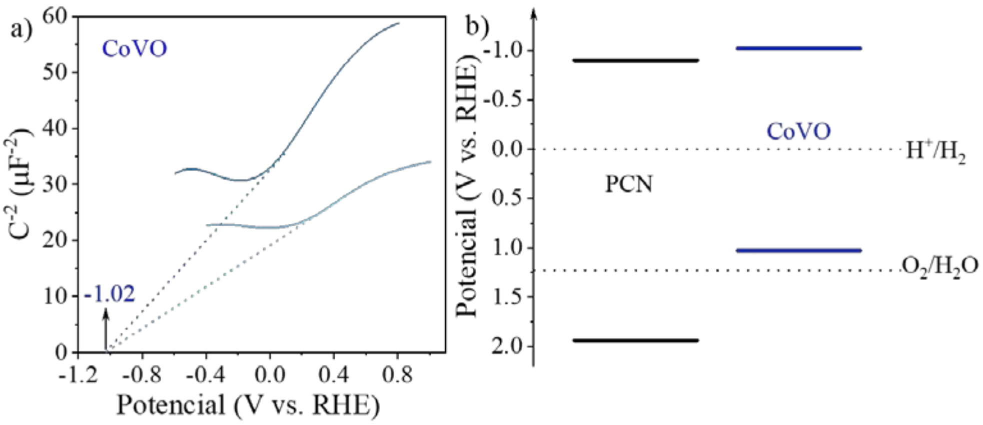

Figure S7. (a) Mott–Schottky plots of CoVO; (b) electronic band structure of the PCN and CoVO.
